# Induction of Apoptosis in Human Pancreatic Cancer Stem Cells by the Endoplasmic Reticulum-Targeted Alkylphospholipid Analog Edelfosine and Potentiation by Autophagy Inhibition

**DOI:** 10.3390/cancers13236124

**Published:** 2021-12-05

**Authors:** Consuelo Gajate, Odile Gayet, Nicolas A. Fraunhoffer, Juan Iovanna, Nelson Dusetti, Faustino Mollinedo

**Affiliations:** 1 Laboratory of Cell Death and Cancer Therapy, Department of Molecular Biomedicine, Centro de Investigaciones Biológicas Margarita Salas, Consejo Superior de Investigaciones Científicas (CSIC), Ramiro de Maeztu 9, E-28040 Madrid, Spain; consuelogajate@gmail.com; 2 Centro de Investigación del Cáncer, Instituto de Biología Molecular y Celular del Cáncer, Campus Miguel de Unamuno, Consejo Superior de Investigaciones Científicas (CSIC)-Universidad de Salamanca, E-37007 Salamanca, Spain; 3 Centre de Recherche en Cancérologie de Marseille (CRCM), INSERM U1068, CNRS UMR 7258, Institut Paoli-Calmettes, Aix-Marseille Université, Parc Scientifique et Technologique de Luminy, CEDEX 09, 13288 Marseille, France; odile.gayet@inserm.fr (O.G.); nicolas.fraunhoffer@inserm.fr (N.A.F.); juan.iovanna@inserm.fr (J.I.); nelson.dusetti@inserm.fr (N.D.)

**Keywords:** pancreatic cancer stem cell, pancreatic cancer spheroid, endoplasmic reticulum, endoplasmic reticulum targeting, autophagy, pancreatic cancer, primary cultures from pancreatic cancer patients, alkylphospholipid analog, antitumor ether lipid, edelfosine

## Abstract

**Simple Summary:**

Pancreatic cancer remains an incurable cancer with a gloomy prognosis and a median survival of months since the time of diagnosis. Disappointingly, no significant improvement in clinical outcomes has been achieved for the last fifty years. Cancer stem cells (CSCs) are suggested to be critical and responsible for tumor development, drug resistance and cancer relapse, therefore, an effective antitumor therapy against pancreatic cancer should deal with pancreatic CSCs. Here, we found that the alkylphospholipid analog edelfosine induces apoptosis in pancreatic CD44^+^CD24^+^EpCAM^+^ CSCs through its accumulation in the endoplasmic reticulum, leading to sustained endoplasmic reticulum stress and cell death. Edelfosine was effective against primary cultures from pancreatic cancer patients and their corresponding pancreatic CSCs. Autophagy inhibition potentiated the proapoptotic action of edelfosine in pancreatic CSCs. Thus, endoplasmic reticulum targeting and its combination with autophagy inhibitors could open a new framework in pancreatic cancer chemotherapy, directly involving pancreatic CSCs.

**Abstract:**

Pancreatic cancer is one of the most lethal malignancies with a poor and gloomy prognosis and the highest mortality-to-incidence ratio. Pancreatic cancer remains an incurable malignancy, and current therapies are ineffective. We isolated cancer stem cells (CSCs) from the human PANC-1 pancreatic cancer cell line as CD44^+^CD24^+^EpCAM^+^ cells. These CSCs form pancreatic cancer spheres or spheroids and develop tumors in SCID mice after subcutaneous injection of as few as 100 cells per mouse. Here, we found that the alkylphospholipid analog edelfosine inhibited CSC pancreatic cancer spheroid formation and induced cell death, as assessed by an increase in the percentage of cells in the sub-G_0_/G_1_ region by means of flow cytometry, indicative of DNA breakdown and apoptosis. This correlated with an increase in caspase-3 activity and PARP breakdown, as a major substrate of caspase-3, following PANC-1 CSC treatment with edelfosine. The antitumor ether lipid edelfosine colocalized with the endoplasmic reticulum in both PANC-1 cells as well as PANC-1 CSCs by using a fluorescent edelfosine analog, and induced an endoplasmic reticulum stress response in both PANC-1 cells and PANC-1 CSCs, with a potent CHOP/GADD153 upregulation. Edelfosine elicited a strong autophagy response in both PANC-1 cells and PANC-1 CSCs, and preincubation of CSCs with autophagy inhibitors, chloroquine or bafilomycin A1, enhanced edelfosine-induced apoptosis. Primary cultures from pancreatic cancer patients were sensitive to edelfosine, as well as their respective isolated CSCs. Nontumorigenic pancreatic human cell line HPNE and normal human fibroblasts were largely spared. These data suggest that pancreatic CSCs isolated from established cell lines and pancreatic cancer patients are sensitive to edelfosine through its accumulation in the endoplasmic reticulum and induction of endoplasmic reticulum stress.

## 1. Introduction

Pancreatic cancer is one of the most aggressive and lethal types of malignant tumors, with an annual incidence rate almost identical to the mortality rate [1]. In 2020, ~495,000 people worldwide were diagnosed with this fatal disease, and ~466,000 patients died of this cancer [2]. Pancreatic ductal adenocarcinoma (PDAC) represents the vast majority (90–95%) of pancreatic cancers and features a dismally poor prognosis, with a median survival of months and a 5-year survival of less than 10% [3,4,5]. Pancreatic cancer remains an incurable cancer, and clinical outcomes have not improved substantially over the past five decades, with no substantial changes in the survival rate [1,5,6,7]. Despite the development of novel treatments, such as FOLFIRINOX, nab-paclitaxel/Abraxane plus gemcitabine and different drug combinations, including gemcitabine, cisplatin, temsirolimus and bevacizumab, their modest effectiveness leading to very poor clinical outcomes, development of chemoresistance and occurrence of significant adverse effects have prevented the achievement of an effective therapy against this grim tumor [1].

There is currently a notion that the capacity of cancer growth and relapse is highly dependent on the presence of viable cancer stem cells (CSCs), which constitute a small subset of cells in the tumor (less than 5% of the total tumor cells) and are critical for cancer initiation, development, invasion, metastasis and drug resistance [8,9,10,11,12]. This CSC population represents a reservoir of self-sustaining cells that can regenerate all facets of the tumor, giving rise to self-renewed CSCs as well as more differentiated cells that comprise the bulk of the tumor population [13], in part through deregulated RNA processing [14]. CSCs show an intrinsic resistance against chemotherapy and radiation [15,16,17], and therefore these latter treatments could eliminate the bulk of non-CSC differentiated tumor cells, but not CSCs [18]. Thus, CSCs, due to their endogenous resistance mechanisms and ability to self-renew and differentiate into heterogeneous lineages of cancer cells, are suggested to underlie drug resistance and cancer relapse, thus leading eventually to more aggressive tumor recurrence and metastasis, the latter being the cause of more than 90% of cancer-related deaths [19]. Pancreatic CSCs were first reported by the laboratory of Diane M. Simeone [20], showing that a CD44^+^CD24^+^EpCAM^+^ (epithelial cell adhesion molecule)/ESA^+^ (epithelial specific antigen) subpopulation, accounting for 0.2–0.8% of pancreatic cancer cells from primary human tumors, was able to display stem cell features of self-renewal, generate differentiated progeny and exhibited a high potential to form tumors in immunocompromised mice (approximately 100-fold increased tumorigenic potential as compared to non-triple-positive cancer cells).

The antitumor ether lipid edelfosine (1-*O*-octadecyl-2-*O*-methoxy-*rac*-glycero-3-phosphocholine) is the prototype of a family of synthetic compounds collectively known as alkylphospholipid analogs (APLs) [21,22,23,24], and is able to induce a potent proapoptotic activity against hematological and solid tumors through a unique mechanism of action that involves lipid rafts and the endoplasmic reticulum (ER) [25,26,27,28,29,30,31,32,33]. Edelfosine also displays in vivo antitumor activity following oral administration in immunodeficient mouse xenograft models for several hematological and solid tumors [29,31,34,35]. Edelfosine shows a rather selective proapoptotic action against tumor cells, whereas normal nonmalignant cells are spared [21,22,26,27,36,37]. Oral administration of edelfosine in mouse xenograft models of pancreatic cancer led to tumor regression and apoptosis in cancer cells [29]. In vitro and in vivo experiments indicated that edelfosine accumulated in the ER of pancreatic cancer cells, leading to ER stress and apoptosis [29]. It is worth noting that pancreatic cells have a very high capacity for protein synthesis because they are specialized cells for the generation of digestive enzymes and hormones, and accordingly they exhibit a highly developed ER [1,38]. Thus, we proposed ER targeting as a novel and effective approach to pancreatic cancer therapy [1,29].

Because CSCs are suggested as the origin of cancers and considered to be responsible for drug resistance and cancer relapse, due to their protective mechanisms against chemotherapy and radiation, they should be the prime targets for therapeutic intervention [39,40,41]. In this way, targeting and getting rid of CSCs may have the potential to cure cancer.

Here, we investigated the action of edelfosine on human pancreatic CSCs derived from the established PANC-1 human pancreatic cancer cell line as well as from primary cultures of pancreatic cancer patients. We also analyzed the role of ER and autophagy in the proapoptotic activity of the antitumor ether lipid edelfosine as a novel, selective and promising anticancer approach against this incurable cancer for which there is no effective treatment.

## 2. Materials and Methods

### 2.1. Reagents

Edelfosine was obtained from R. Berchtold (Biochemisches Labor, Bern, Switzerland), and stock solutions were prepared as previously described [36]. Edelfosine was dissolved at 2 mM, as a stock solution, in the culture medium containing 10% (*v*/*v*) heat-inactivated fetal bovine serum (FBS) by heating at 50 °C for 45 min. The clear solution was sterilized by filtration through a 0.22 μm pore size Millipore (Millipore Corporation, Burlington, MA, USA) sterile filter and stored at 4 °C until use. The pan-caspase inhibitor z-VAD-fmk was from Alexis Biochemicals (San Diego, CA, USA). Chloroquine and bafilomycin A1 were from Sigma-Aldrich (St. Louis, MO, USA).

### 2.2. Cell Culture

The human pancreatic cancer cell line PANC-1 was obtained from the American Type Culture Collection (ATCC, Manassas, VA, USA). Monolayer cultures of PANC-1 (approximate doubling time, 32 h) were grown in Dulbecco’s modified Eagle’s medium (DMEM) supplemented with 10% (*v*/*v*) heat-inactivated FBS, 2 mM L-glutamine, 100 U/mL penicillin, and 100 μg/mL streptomycin at 37 °C in a humidified atmosphere containing 5% CO_2_. Ciprofloxacin (12.5 μg/mL) was added to avoid mycoplasma contamination. The cells were passaged at 80–90% confluence using Versene solution (GIBCO-BRL, Gaithersburg, MD, USA). The cells were periodically tested for mycoplasma infection and found to be negative.

The nontumorigenic hTERT (human telomerase reverse transcriptase)-immortalized human pancreatic nestin-expressing (HPNE) cell line, developed from human pancreatic duct by transduction with a retroviral expression vector containing the hTERT gene [42], was purchased from ATCC. Normal human primary fibroblasts were isolated from hypertrophic tonsils as previously described [37]. HPNE and fibroblasts were grown in DMEM culture medium supplemented with 10% (*v*/*v*) heat-inactivated FBS as above.

### 2.3. Pancreatic Cancer Spheroid Formation

PANC-1 cells were grown in DMEM containing 10% FBS in 75 cm^2^ cell culture flasks till 80–90% confluence was reached. Then, after two washes with Dulbecco’s phosphate-buffered saline (DPBS) (GIBCO, Carlsbad, CA, USA), without calcium and magnesium, approximately 1.2 × 10^6^ pancreatic cancer cells were seeded in a serum-free stem cell medium containing DMEM/F12, B27 (2% (*v*/*v*) of 50× B27), 20 ng/mL recombinant human epidermal growth factor (rhEGF) (Invitrogen, Waltham, MA, USA), 20 ng/mL recombinant human fibroblast growth factor (FGF) (Invitrogen), 2 mM L-glutamine, 100 U/mL penicillin, 100 μg/mL streptomycin, using ultralow attachment 75 cm^2^ canted cell culture flasks with a vent cap (Corning Life Sciences, Tewksbury, MA, USA). The above culture medium was referred to as the “cancer stem cell medium” (CSM). The cells were cultured at 37 °C in a humidified atmosphere containing 5% CO_2_. The medium was changed every 4–5 days until pancreatic cancer spheroids of an appropriate size were obtained. After the primary pancreatic cancer spheroids reached approximately 100–200 μm in diameter, the spheres were dissociated and single cells were cultured in CSM to form secondary spheroids. Pancreatic cancer spheres were subcultured several times for at least seven generations. After the pancreatic cancer spheroids reached the desired size, cancer sphere cultured cells were disaggregated to a single-cell suspension using the StemPro^®^ Accutase^®^ Cell Dissociation Reagent (GIBCO) according to the manufacturer’s specifications.

### 2.4. Sorting of Cancer Stem Cells by Means of Flow Cytometry

Pancreatic cancer spheroids (usually approximately 100–200 μm in size) were collected with a 40 μm BD Falcon cell strainer. Then, pancreatic cancer spheroids were disaggregated using the StemPro^®^ Accutase^®^ Cell Dissociation Reagent (GIBCO), washed twice with DPBS and suspended in DPBS at 1 × 10^6^ cells/100 μL. Then, the cells were sorted for CD44^+^CD24^+^EpCAM^+^ pancreatic CSCs using anti-CD44-APC (1:40 dilution), anti-CD24-PE (1:40 dilution) and anti-EpCAM-FITC (1:40 dilution) antibodies (Becton Dickinson, Franklin Lakes, NJ, USA) and a FACSAria cell sorter (Becton Dickinson) equipped with 488 nm (blue) and 633 nm (red) emitting lasers. Side-scatter and forward-scatter profiles were used to eliminate cell doublets. The data were analyzed with the BD FACSDiva software.

### 2.5. Soft Agar Colony Assay

Cell suspensions (5 × 10^3^ cells) from disaggregated pancreatic cancer spheroids suspended in CSM were prepared at 1.5 mL/well with 0.3% agarose (Becton Dickinson) in the presence or absence of edelfosine, and overlaid onto each well of a six-well plate containing a solidified bottom layer of 0.6% agarose in the medium (2 mL/well). Once the top layer solidified, 1 mL of CSM was placed on top of the cell layer, and the plates were incubated for 3 weeks, and the colonies were counted using a stereo microscope Leica MZ16F (Wetzlar, Germany). The colony size was evaluated by measuring the colony perimeter using the ImageJ software (NIH, Bethesda, MD, USA). Vehicle-treated control cells were run in parallel. The results are expressed as the mean arbitrary units ± SD from three independent experiments in two replicates.

### 2.6. Xenograft Mouse Models

Animal procedures were approved by the institutional research commission of the University of Salamanca and the Ethics Committee of the University of Salamanca. Animal procedures complied with the Spanish (Real Decreto RD1201/05) and the European Union’s (European Directive 2010/63/EU) guidelines on animal experimentation for the protection and humane use of laboratory animals, and were conducted at the accredited Animal Experimentation Facility (Servicio de Experimentación Animal) of the University of Salamanca (register No. PAE/SA/001). Female CB17 severe combined immunodeficient (SCID) mice (8-week-old) (Charles River Laboratories, Lyon, France), kept and handled according to the institutional guidelines complying with the Spanish legislation under the 12/12 h light/dark cycle at a temperature of 22 °C, received a standard diet and acidified water ad libitum. Different numbers of sorted CD44^+^CD24^+^EpCAM^+^ pancreatic CSCs (10^2^, 10^3^ and 10^4^) were suspended in the serum-free CSM/Matrigel mixture (1:1 volume) followed by subcutaneous injection into the right flank of each mouse. For comparison, similar amounts of PANC-1 cells were also injected in immunodeficient mice as previously described [29]. The animals were monitored for body weight and any signs of morbidity daily. The animals were sacrificed 4 months after cancer cell implantation; the tumors were then carefully removed, and tumor weight and volume were measured.

### 2.7. Apoptosis Analysis by Means of Flow Cytometry

Quantification of apoptotic cells was determined by means of flow cytometry with a Becton Dickinson FACSCalibur and a Beckman-Coulter (Brea, CA, USA) FC500-MPL flow cytometers as the percentage of cells in the sub-G_0_/G_1_ region (hypodiploid cells) in cell cycle analysis as previously described [37,43,44].

### 2.8. Confocal Microscopy of Pancreatic Cancer Spheroids

Pancreatic cancer spheroid cells were attached to slides via cytospin at low speed (Shandon Cytospin 3; Shandon Scientific, Runcorn, Cheshire, UK) and then fixed in 4% formaldehyde using a Coplin jar as previously described [45]. The presence of CD44, CD24 and EpCAM was examined by means of confocal microscopy using mouse anti-human CD44 conjugated to phycoerythrin, mouse anti-human CD24 conjugated to APC and mouse anti-human EpCAM conjugated to FITC monoclonal antibodies (1:10 dilution) (Becton Dickinson) as previously described [45,46]. To examine the presence of PAX6 and PDX1, the cells were incubated with the rabbit polyclonal antibody to PAX6 (Abcam, Cambridge, UK) and the mouse monoclonal antibody to PDX1 (Abcam) as the primary antibodies (1:10 dilution) and the goat anti-rabbit IgG Cy3-conjugated and anti-mouse IgG FITC-conjugated secondary antibodies, respectively. Fluorescence was visualized with a Zeiss LSM 310 confocal laser scanning microscope (Zeiss, Oberkochen, Germany).

### 2.9. Analysis of Subcellular Edelfosine Localization by Confocal Microscopy

The subcellular localization of edelfosine in pancreatic cancer spheroids was examined with the edelfosine fluorescent analog 1-*O*-[11〲-(6〳-ethyl-1〳,3〳,5〳,7〳-tetramethyl-4〳,4〳-difluoro-4″-bora-3a″,4a″-diaza-*s*-indacen-2〳-yl)undecyl]-2-*O*-methyl-*rac*-glycero-3-phosphocholine (Et-BDP-ET) [47], which contains a boron-dipyrromethene (BODIPY) molecule, a kind gift from F. Amat-Guerri and A.U. Acuña (Consejo Superior de Investigaciones Científicas, Madrid, Spain). ER was visualized using the CellLight™ ER red fluorescent protein (RFP) BacMam 2.0 reagent (ThermoFisher, Waltham, MA, USA), following the manufacturer’s specifications. This CellLight™ ER-RFP BacMam 2.0 reagent includes a fusion construct of the ER signal sequence of calreticulin and KDEL (ER retention signal) and TagRFP, providing accurate and specific targeting to cellular ER-RFP, with minimal cellular disruption. Colocalization of both signals (RFP; excitation: 555 nm, emission: 574–627 nm; BODIPY; excitation: 498 nm, emission: 510–549 nm) was analyzed by excitation of both fluorochromes in the same section. Fluorescence was visualized with a confocal laser scanning microscope Leica TCS SP8 STED 3X fitted with a 405 nm UV laser and a white-light laser with freely tunable excitation (470–670 nm).

### 2.10. Western Blotting

The cells were harvested in a lysis buffer containing 25 mM HEPES (pH 7.7), 0.3 M NaCl, 1.5 mM MgCl_2_, 0.2 mM EDTA, 0.1% Triton X-100, 20 mM β-glycerophosphate, 0.1 mM sodium orthovanadate, supplemented with protease inhibitors (1 mM phenylmethylsulfonyl fluoride, 20 μg/mL aprotinin, 20 μg/mL leupeptin) or a protease inhibitor cocktail (Roche, Basel, Switzerland). Protein concentration was determined using a Bradford assay. Proteins (30–40 μg) were run on SDS-PAGE (8%, 10% or 14%) and analyzed by means of immunoblotting. The separated proteins were transferred to Immobilon-P polyvinylidene difluoride (PVDF) or nitrocellulose membranes, and then the blots were blocked with 5% (*w*/*v*) dried skimmed milk powder in 50 mM Tris HCl (pH 8.0), 150 mM NaCl and 0.1% Tween 20 (TBST) for 60 min at room temperature, and incubated for 1 h at room temperature or overnight at 4 °C with specific antibodies. The following primary antibodies used for protein immunodetection in this study are listed below: anti-116 kDa poly(ADP-ribose) polymerase (PARP) (1:1000 dilution) mouse monoclonal antibody that also detects the p85 cleavage fragment (BD Biosciences Pharmingen, San Diego, CA, USA); anti-32 kDa caspase-3 (1:1000 dilution) rabbit monoclonal antibody that also detects the p20 and p17 subunits (BD Biosciences Pharmingen); anti-16–14 kDa LC3 (1:1000 dilution) rabbit polyclonal antibody (Cell Signaling Technology, Danvers, MA, USA); anti-30 kDa CHOP/GADD153 (1:250 dilution) rabbit polyclonal antibody (Santa Cruz Biotechnology); anti-125 kDa PERK (1:500 dilution) rabbit polyclonal antibody (Santa Cruz Biotechnology); anti-125 kDa phosho-PERK (1:500 dilution) rabbit polyclonal antibody (Santa Cruz Biotechnology); anti-78 kDa GRP78/BiP (1:1000 dilution) rabbit polyclonal antibody (Santa Cruz Biotechnology); anti-42/44 kD p-ERK1/2 (1:1000 dilution) mouse monoclonal antibody (Cell Signaling Technology); anti-42 kDa β-actin (1:5000 dilution) mouse monoclonal antibody (Sigma). After three 5 min TBST washes, the membranes were incubated with horseradish peroxidase (HRP)-conjugated secondary antibodies (1:1000 dilution) for 1 h at room temperature. Then, the protein bands were visualized with a ChemiDoc^TM^ Imaging System (Bio-Rad, Hercules, CA, USA), or by means of autoradiography, using an enhanced chemiluminescence detection kit Amersham ECL (GE Healthcare, Chicago, IL, USA).

Images of the immunoblots were captured using the ChemiDoc Imaging System or after scanning developed autoradiography films, and then quantification of the relative level of the protein bands with reference to the internal control β-actin was calculated using the ImageJ software [48] as previously described [49]. Data were acquired as arbitrary units, which were then normalized against the internal control β-actin and expressed as values relative to the untreated control. Original blots are shown in the Supplementary Materials (Figure S1).

### 2.11. Primary Cultures from Pancreatic Cancer Patients

Fresh tumor samples from patients included under the PaCaOmics clinical trial (ClinicalTrials.gov: NCT01692873) were used to generate patient-derived xenografts (PDX), from which primary cell cultures were derived as described [50]. This study was approved by the Paoli-Calmettes hospital ethics committee following informed consent of the patients.

All the experiments on mice were performed after approval by the ethics committee for animal experimentation and the French Ministry of Higher Education and Research (APAFIS No. 9562-2016051914513578, version 4). The samples were mixed with 100 µL Matrigel and implanted subcutaneously in immunodeficient mice. The mice were sacrificed when PDX reached a volume of 1 cm^3^. Xenografts were minced and treated with collagenase type V (Sigma-Aldrich) and trypsin/EDTA (GIBCO). The cells were suspended in DMEM supplemented with 1% *w*/*w* penicillin/streptomycin (GIBCO, Life Technologies) and 10% heat-inactivated FBS (Lonza Inc., Walkersville, MD, USA). After centrifugation, the cells were seeded in a serum-free ductal medium (SFDM), adapted from Schreiber et al. [51], and incubated at 37 °C in a 5% CO_2_ incubator. The final concentrations of SFDM included: 1.22 mg/mL nicotinamide, 5 mg/mL glucose, 5% insulin–transferrin–selenium (ITS+ containing bovine serum albumin and linoleic acid), 100 μg/mL gentamicin, 5% NuSerum IV, 25 μg/mL bovine pituitary extract (BPE), 20 ng/mL EGF, 50 nM 3,3′,5-triiodo-L-thyronine (T3), 1 μM dexamethasone, 100 ng/mL cholera toxin, 1× penicillin/streptomycin and 1× antibiotic–antimycotic (anti/anti) (GIBCO) (the latter only during the first week of culture from PDX). The differences from Schreiber’s experimental conditions [51] included a higher concentration of T3 (50 nM instead of 5 nM), use of penicillin/streptomycin and anti/anti solutions.

### 2.12. Chemograms

Chemograms with edelfosine were performed as described in [50]. Briefly, five thousand cells were plated per well in 96-well plates in SFDM [51]. Twenty-four hours later, increasing concentrations of edelfosine ranging from 1 nM to 50 µM were added. Each experiment was performed twice in triplicate. After a 72 h treatment, cell viability was estimated with the addition of the PrestoBlue Reagent (Thermo Fisher, Waltham, MA, USA) for 3 h according to the protocol provided by the supplier and quantified using plate reader Tristar LB941 (Berthold Technologies, Bad Wildbad, Germany). In the case of CSCs, colorimetric assays using XTT were carried out as previously described [52], incubating growing cells in 96-well flat-bottomed microtiter plates at 37 °C in a humidified atmosphere of 5% CO_2_/95% air for 72 h, with different concentrations of the assayed compounds. Measures to quantify the cell response to drugs were processed using the GRmetrics R package [53] to calculate the growth rate (GR) inhibition metrics and obtain the fitted curves for each cell culture. The GR metric is defined as the ratio between the growth rates of the treated cell and the untreated cell in a base-2 exponential function minus one, which falls between −1 and 1, where the GR values in the range from 1 and 0 are partially cytostatic and those below 0 are cytotoxic, whereas the values above 1 indicate a higher growth rate in the treated cells than in the untreated cells. GR_50_ is defined as the concentration of a drug that reduces cell growth by half [54], and measures the effect of a perturbation on the growth rate of a cell population. Each GR_50_ value represents the mean of three independent determinations performed in triplicate.

### 2.13. Statistical Analysis

The data are shown as the means ± SD of the number of experiments indicated. Statistical evaluation was performed using Student’s *t*-test. A *p*-value < 0.05 was considered to be statistically significant.

## 3. Results

### 3.1. Isolation and Characterization of Human Pancreatic CSCs from the PANC-1 Cell Line

**Figure 1:**
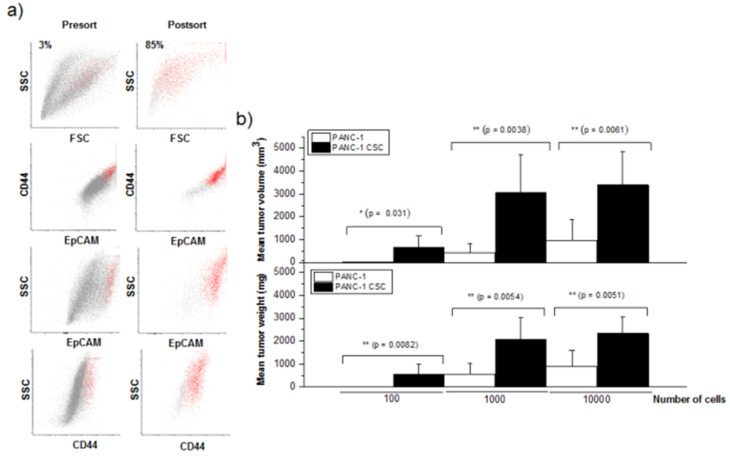
Sorting of pancreatic cancer stem cells and tumorigenic activity. (**a**) Following disaggregation of PANC-1-derived pancreatic cancer spheres, CD44^+^CD24^+^EpCAM^+^ CSCs were purified by means of flow cytometry sorting as described in the Section 2. The percentage of the cells corresponding to the CSC population is indicated in the pre-sorting (Presort) and post-sorting (Postsort) histograms. (**b**) In immunodeficient mice, 10^2^, 10^3^ and 10^4^ PANC-1 cells and freshly sorted CD44^+^CD24^+^EpCAM^+^ PANC-1 CSCs were injected, and tumor development was analyzed after 4 months. Following 17 weeks, the animals were sacrificed and the weight and volume of each tumor generated in the animals were determined, and the corresponding average values were presented. The data are the means ± SD (*n* = 10); *p* values represent the statistically significant difference in the generated tumor size and weight between the mice injected with PANC-1 cells and CD44^+^CD24^+^EpCAM^+^ PANC-1 CSCs; *, *p* < 0.05; **, *p* < 0.01.

Because pancreatic CSCs can form pancreatic cancer spheroids and express CD44, CD24 and EpCAM/ESA [20,55,56], we first isolated pancreatic CSCs from the human PANC-1 pancreatic cancer cell line, based on their ability to generate pancreatic cancer spheroids in a serum-free medium containing well-defined growth factors and their enrichment in CD44, CD24 and EpCAM (Figure 1a). PANC-1 cells generated pancreatic cancer spheroids of approximately 100–200 μm in diameter after approximately 3–4 weeks under specific culture conditions, and grew throughout the incubation time. Pancreatic cancer spheres were mechanically dissociated into single cells in a special cell disaggregation medium and sorted for CD44^+^CD24^+^EpCAM^+^ cells by means of flow cytometry (Figure 1a). Triple-positive CSCs were enriched to >85% from an approximately 3% triple-positive CSC population before presorting (Figure 1a). Following the sorting, different amounts of the sorted CD44^+^CD24^+^EpCAM^+^ cells were assayed for their capability to generate tumors in immunodeficient mice, and compared to the corresponding tumorigenic activity exerted by the same amounts of unsorted PANC-1 cells. Sorted cells were suspended in the Matrigel mixture (1:1, *v*/*v*) and injected subcutaneously into the right flank of SCID mice. Tumor growth was monitored every week for 17 weeks, and then the animals were sacrificed and tumor weight and volume were measured (Figure 1b). The number of mice that generated tumors is shown in Table 1. Tumors developed in 6 out of 10 animals following injection of 100 CD44^+^CD24^+^EpCAM^+^ cells and in 10 out of 10 animals after injection of 10^3^ or 10^4^ CD44^+^CD24^+^EpCAM^+^ cells (Table 1). However, no tumors developed after injection of 100 parental PANC-1 cells (Table 1). CD44^+^CD24^+^EpCAM^+^ CSCs were able to generate tumors when as few as 100 cells were transplanted in approximately 60% of the immunocompromised mice (Table 1 and Figure 1b), whereas an injection of 10^4^ PANC-1 cells was required to form tumors in immunodeficient mice in a similar percentage (Table 1), suggesting that PANC-1 CSCs had a 100-fold increased tumorigenic potential as compared to the parental PANC-1 cells. The size of the tumors was always significantly higher in the mice injected with PANC-1 CSCs than in the animals injected with parental PANC-1 cells (Figure 1b).

**Figure 2:**
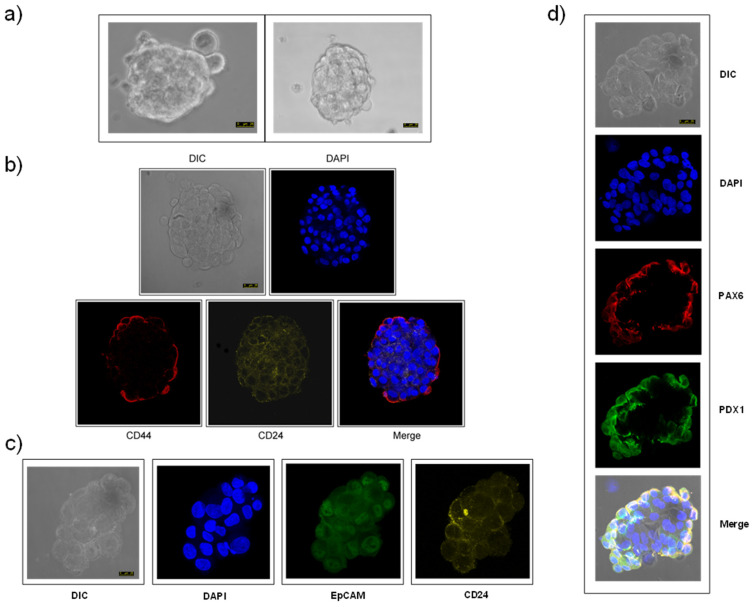
CSC pancreatic cancer spheroids and expression of pancreatic CSC markers. Pancreatic cancer spheroids were generated from human pancreatic PANC-1 cancer cells as described in the Section 2, and the corresponding differential interference contrast (DIC) microscopy images are shown (**a**). Pancreatic cancer spheroids were analyzed for the expression of CD44 (**b**), CD24 (**b**,**c**), EpCAM (**c**), PAX (**d**) and PDX1 (**d**) by confocal microscopy as described in the Section 2. DIC images are also shown. The cells were also stained for nuclei with 4′,6-diamidino-2-phenylindole (DAPI) (blue fluorescence). Bar, 25 μm. Data are representative of at least three independent experiments.

The sorted PANC-1 CSCs generated pancreatic cancer spheres of approximately 100–200 μm in diameter rapidly following only ~5-day incubation under specific culture conditions in a serum-free cancer stem cell medium (Figure 2a), and grew throughout the incubation time. Confocal microscopy revealed that these PANC-1 CSC pancreatic cancer spheroids were labeled with antibodies against CD44, CD24, EpCAM (Figure 2b,c). PANC-1 CSC pancreatic cancer spheroids also expressed PAX6, a transcription factor involved in pancreatic cancer and the maintenance of stem cells [57,58,59], and pancreatic and duodenal homeobox 1 (PDX1), also known as insulin promoter factor 1 (IPF1), a key transcription factor in pancreatic embryogenesis and pancreatic cell differentiation [60,61], as assessed by confocal microscopy (Figure 2d). CSC pancreatic cancer spheres were sorted by CD44–CD24–EpCAM both before plating and after sphere formation for the different assays shown in this study. Interestingly, the PANC-1 CSC pancreatic cancer spheroids could be subcultured several times for at least seven generations, and these CSC pancreatic cancer spheres, again, contained only 2.8–7.5% of CD44^+^CD24^+^EpCAM^+^ cells after prolonged culture. This supports and highlights the stemness of the herein generated pancreatic CSCs, because they are able to self-renew in triple-positive CSCs and differentiate into more heterogeneous and mature cancer cells.

### 3.2. Edelfosine Inhibits Formation of CSC Pancreatic Cancer Spheroids

**Figure 3:**
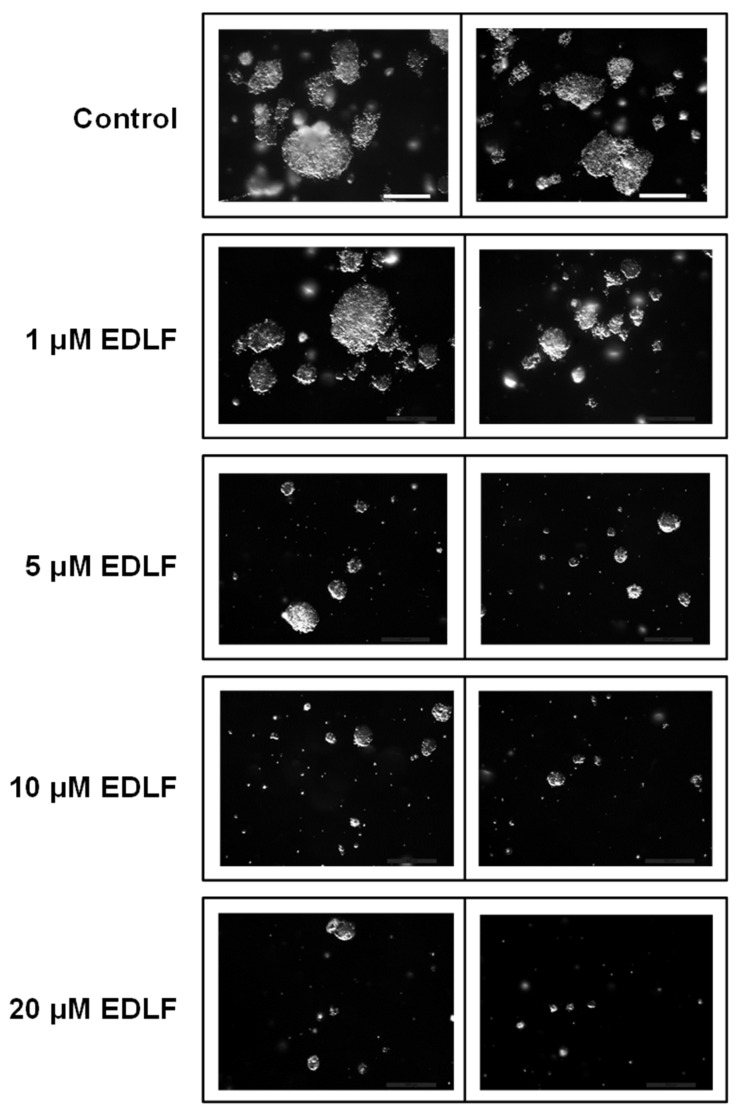
Effect of edelfosine on the formation of PANC-1 CSC pancreatic cancer spheroids. Disaggregated CSCs from PANC-1 CSC pancreatic cancer spheroids were assayed for the formation of colonies in soft agar analysis in the absence and in the presence of different concentrations of edelfosine (EDLF). Bar, 600 μm. The data are representative of at least three independent experiments.

We next examined the effect of edelfosine on pancreatic cancer spheroid formation. To this aim, pancreatic PANC-1 CSCs, after being sorted by CD44–CD24–EpCAM as above, were grown in soft agar using stem cell culture conditions and treated with various concentrations of edelfosine for 21–28 days. As shown in Figure 3 and Table 2, edelfosine effectively and significantly inhibited pancreatic cancer spheroid formation at 5 μM, and the inhibition was very potent when the antitumor ether lipid was used at 10 or 20 μM. Likewise, the number of PANC-1 CSC spheroid colonies was highly reduced following incubation with ≥ 5 μM edelfosine (Figure 3 and Table 2), suggesting that edelfosine was able to affect spheroid formation at the low micromolar range by significantly impairing the self-renewal ability of CSCs.

### 3.3. Edelfosine Induces Apoptosis in PANC-1 CSCs

**Figure 4:**
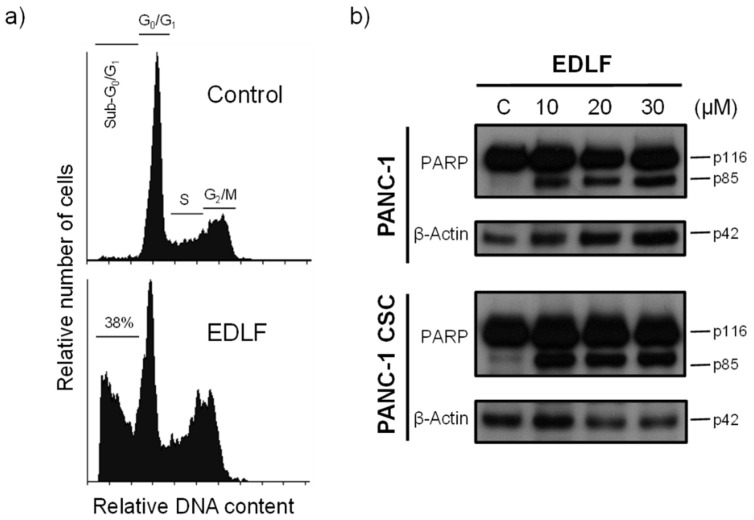
Induction of apoptosis in PANC-1 CSCs by edelfosine. (**a**) PANC-1 CSCs were incubated in the absence (control) or presence of 20 μM edelfosine (EDLF) for 5 days, and then apoptosis was analyzed by means of flow cytometry. The different cell cycle stages are indicated. The percentage of apoptotic cells in the sub-G_0_/G_1_ region is shown. The data are representative of at least three independent experiments. (**b**) PANC-1 cells and PANC-1 CSCs were untreated (C) or treated with edelfosine (EDLF) at the indicated concentrations for 48 h and analyzed by means of Western blotting using specific antibodies for PARP; β-actin was used as the loading control. Molecular weights (in kilodaltons) of every protein are indicated at the right side of each panel. The gels were cropped to show the relevant sections. The Western blot images are representative of three independent experiments.

We previously found in animal models that the pharmacologically relevant and effective concentration of edelfosine in plasma ranges between 10 and 20 μM [34,35,62]. After incubating PANC-1 CSC pancreatic cancer spheroids with 20 μM edelfosine for 5 days, we found induction of an apoptotic response (~38% apoptosis), as assessed by an increase in the percentage of cells in the sub-G_0_/G_1_ region, indicative of DNA breakdown (Figure 4a). Parental PANC-1 cells were also sensitive to edelfosine under similar experimental conditions (~47% apoptosis, after 5-day incubation). This apoptotic response was inhibited (~90% inhibition) by the pan-caspase inhibitor z-VAD-fmk (37.6 ± 9.2% and 46.7 ± 8.3% apoptosis following treatment of PANC-1 CSCs and PANC-1 cells with 20 μM edelfosine for 5 days in the absence of the caspase inhibitor versus 5.1 ± 2.1% and 4.9 ± 1.8% apoptosis in the presence of 50 μM z-VAD-fmk, respectively). Further biochemical confirmation of the induction of apoptosis by edelfosine was obtained through the cleavage of the intact 116 kDa poly(ADP-ribose) polymerase (PARP) into the 85 kDa PARP fragment following incubation of PANC-1 and PANC-1 CSCs with different concentrations of edelfosine (Figure 4b), as an early marker of apoptosis [29].

### 3.4. Edelfosine Accumulates in the ER of Parental PANC-1 and PANC-1 CSCs and Induces an ER Stress Response

**Figure 5:**
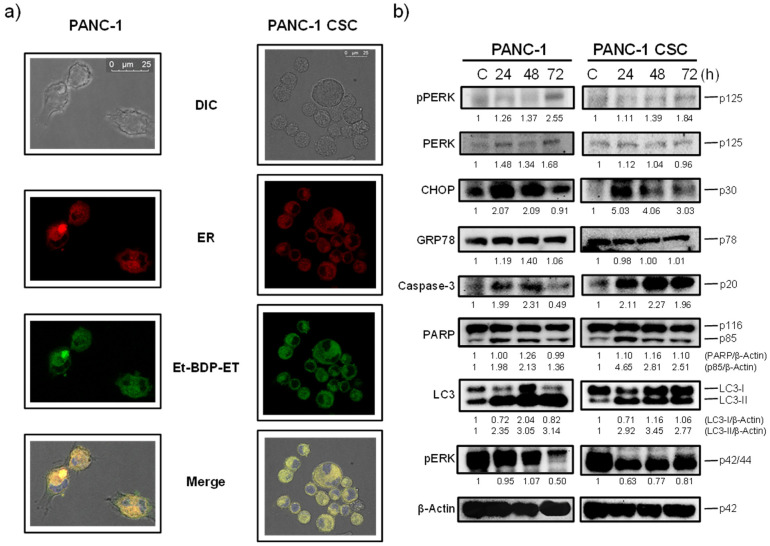
Subcellular localization of edelfosine in the ER of PANC-1 cells and PANC-1 CSC pancreatic cancer spheroids, and induction of an ER stress response. (**a**) Live PANC-1 cells and PANC-1 CSC pancreatic cancer spheroids were labeled overnight at 37 °C for the ER in red using the CellLight ER-RFP BacMam 2.0 reagent, and then the samples were incubated with 20 μM Et-BDP-ET (green fluorescence) for 3 h at 37 °C. Areas of colocalization between the ER and Et-BDP-ET in the merge panels are yellow. The cells were also stained for nuclei with DAPI (blue fluorescence). The corresponding differential interference contrast (DIC) microscopy images are also shown. Bar, 25 μm. The data are representative of three independent experiments. (**b**) PANC-1 cells and PANC-1 CSCs were untreated (C) or treated with 20 μM edelfosine for the indicated times and analyzed by means of Western blotting using specific antibodies for the denoted proteins; β-actin was used as the loading control. Molecular weights (in kilodaltons) of every protein are indicated at the right side of each panel. The gels were cropped to show the relevant sections. Relative protein level quantification after normalization to the internal control β-actin is included below each Western blot panel. The Western blot images are representative of three independent experiments.

Our previous data indicated that edelfosine was taken up by distinct pancreatic cancer cells, including HuP-T4 and Capan-2 human pancreatic cancer cell lines, and accumulated in the ER [29]. Here, we found that parental PANC-1 cancer cells as well as PANC-1 CSCs accumulated the fluorescent edelfosine analog Et-BDP-ET [1-*O*-[11′-(6〳-ethyl-1〳,3〳,5〳,7〳-tetramethyl-4〳,4〳-difluoro-4″-bora-3a″,4a″-diaza-*s*-indacen-2〳-yl)undecyl]-2-*O*-methyl-*rac*-glycero-3-phosphocholine] [47] in the ER (Figure 5a), labeled with a baculovirus-based reagent using the ER signal sequence of calreticulin and KDEL (see Materials and Methods). This fluorescent edelfosine analog has previously behaved as a reliable reagent to visualize the subcellular location of the drug in situ [29,47,63], and its cell incorporation was blocked by adding higher amounts of the parent and unlabeled drug edelfosine.

Edelfosine also induced ER stress in both parental PANC-1 cells and PANC-1 CSCs, as assessed by the phosphorylation of the ER stress marker PERK and the potent upregulation of the C/EBP-homologous protein (CHOP) (also known as growth arrest and DNA damage-inducible gene 153, GADD153, or DNA damage-inducible transcript 3, DDIT3), which activates transcription of the genes potentiating apoptosis as a result of ER stress [64] (Figure 5b). In contrast, edelfosine did not upregulate the ER chaperone 78 kDa glucose-regulated protein (GRP78; also known as BiP, binding immunoglobulin protein) (Figure 5b), which is linked to cell survival during an ER stress response [65]. Interestingly, a potent autophagic response was also triggered upon edelfosine treatment, as assessed by an increase in the level of microtubule-associated protein 1 light chain 3 (LC3)-II form (Figure 5b). The modification of LC3 from the unconjugated form (LC3-I), which is in the cytosol, to the phosphatidylethanolamine-conjugated form (LC3-II), which targets the autophagosomal membrane, is a major marker of autophagy activation [66]. The induction of apoptosis by edelfosine in both PANC-1 and PANC-1 CSCs is biochemically determined by the activation of caspase-3 through the cleavage of pro-caspase-3 into the p20 active caspase-3 fragment (Figure 5b), and the proteolysis of the caspase-3 substrate PARP (Figure 5b). In addition, edelfosine treatment inhibited high basal phosphorylation of the cell survival extracellular signal-regulated kinase 1/2 (ERK1/2) (Figure 5b) without any apparent change in the total ERK1/2 protein level (Supplementary Materials, Figure S1).

### 3.5. Autophagy Inhibitors Potentiate Edelfosine-Induced Apoptosis in PANC-1 and PANC-1 CSCs

**Figure 6:**
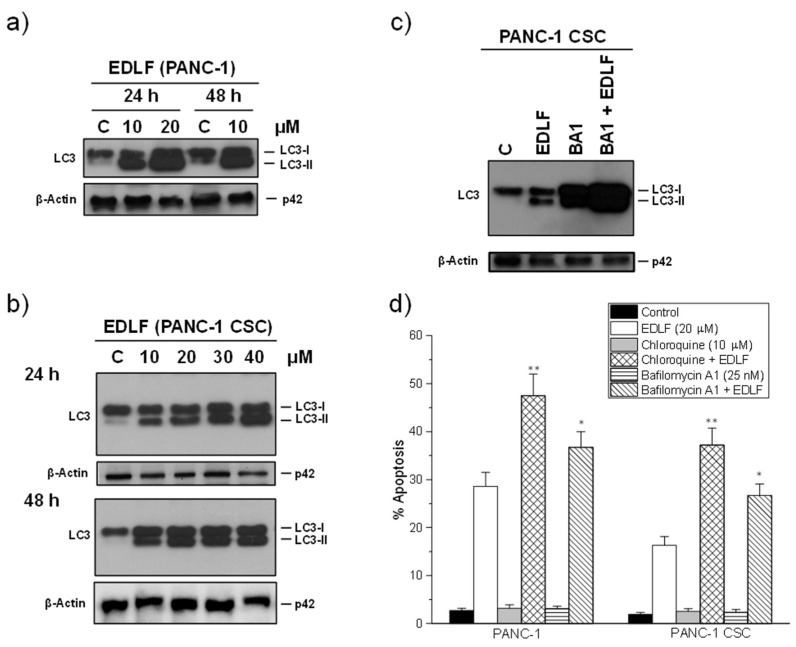
Edelfosine induces autophagy in PANC-1 cells and PANC-1 CSC pancreatic cancer spheroids, and potentiation of cell killing by autophagy inhibition. Autophagy induction was monitored by an increase in the LC3-II band following drug treatment at the indicated drug concentrations and incubation times in both PANC-1 cells (**a**) and PANC-1 CSCs (**b**). (**c**) PANC-1 CSCs were incubated for 48 h in the absence or presence of 20 μM edelfosine (EDLF), 25 nM bafilomycin A1 (BA1) or 25 nM BA1 (1 h preincubation) + 20 μM EDLF; β-actin was used as the loading control in panels (**a**–**c**). The gels were cropped to show the relevant sections. The data are representative of three performed experiments. (**d**) The cells were preincubated for 1 h in the presence or absence of the indicated inhibitors of the autophagic–lysosomal route, and then the cells were incubated in the absence or presence of edelfosine for 72 h. Untreated controls were run in parallel. Apoptosis was then determined by means of flow cytometry as the percentage of cells in the sub-G_0_/G_1_ region. Asterisks indicate significant differences * *p* ˂ 0.05; ** *p* ˂ 0.01. The data shown are the means ± SD of three independent experiments. EDLF, edelfosine.

Because edelfosine induced a potent autophagy response (Figure 5b), and autophagy protects cells against apoptosis induced by different stimuli [67,68,69], being cytoprotective against an ER stress response [70], we examined the effect of autophagy inhibitors on the proapoptotic activity of the antitumor ether lipid. A time course and dose response of the effect of edelfosine on autophagy showed that the alkylphospholipid analog behaved as a potent inducer of autophagy in both PANC-1 and PANC-1 CSCs, with a remarkable and high increase in the level of the LC3-II protein (Figure 6a,b). The incubation of PANC-1 CSCs with the autophagy inhibitor bafilomycin A1, which disrupts the autophagy flux by inhibiting both V-ATPase and autophagosome-lysosome fusion [71], increased dramatically the level of LC3-II following edelfosine treatment (Figure 6c), thus confirming the potent induction of autophagy in CSCs upon edelfosine treatment. Quantification by the ImageJ software of the LC3-II protein band in the Western blot shown in Figure 6c indicated that the LC3-II levels, normalized using the β-actin levels, were increased 15.5, 23.7 and 65.2-fold in relation to the untreated control following incubation with edelfosine, bafilomycin A1 and bafilomycin A1 + edelfosine, respectively. Preincubation with the autophagy inhibitors chloroquine and bafilomycin A1 [71,72] further potentiated edelfosine-induced apoptosis, and this increase was more easily detected in PANC CSCs (Figure 6d). The apoptotic response was inhibited by the pan-caspase inhibitor z-VAD-fmk in ~90% of all the cases, either in the presence or absence of autophagy inhibitors.

### 3.6. Edelfosine Inhibits Cell Proliferation and Induces Cell Death in Patient-Derived Primary Pancreatic Cancer Cell Cultures

To further validate the effect of edelfosine on PDAC, we performed a growth inhibition assay in four PDAC primary cell cultures. Previously, and in order to validate that primary cell cultures are PDAC transformed epithelial cells, 5 × 10^6^ primary cultured cells were subcutaneously implanted in nude mice to obtain PDX. PDX tumors were paraffin-embedded and analyzed by means of hematoxylin–eosin staining and cytokeratin 19 immunostaining (monoclonal rabbit anti-human cytokeratin 19 antibody, Abcam) (Supplementary Materials, Figure S2). Photomicrographs of each primary cell culture used in this study are also shown in the Supplementary Materials (Figure S3). The growth of primary cultured cells as PDX tumors showed that those cells were transformed, and the positive specific staining with anti-human cytokeratin 19 antibodies indicated that those cells were of human epithelial origin.

**Figure 7:**
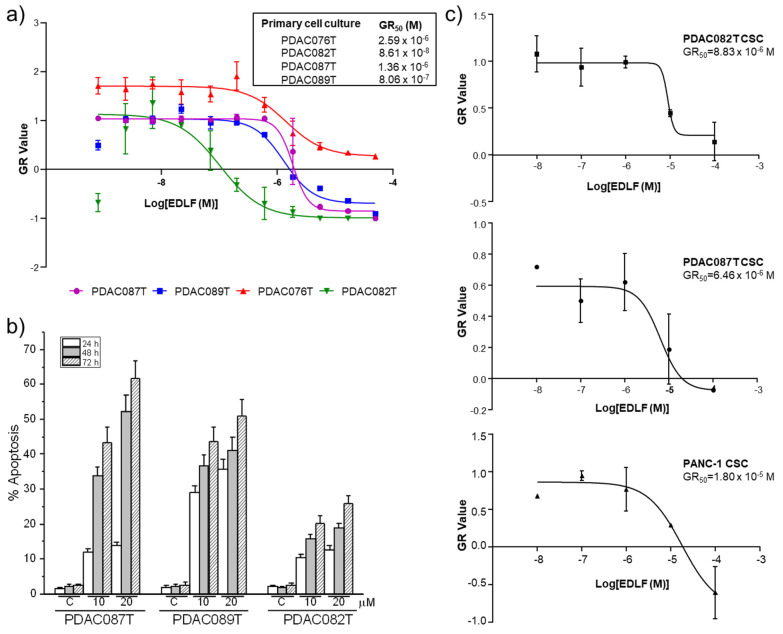
Characterization of the chemosensitivity profile after edelfosine treatment in human pancreatic cancer primary cultures. (**a**) Four different PDAC-derived primary cell cultures were treated with increasing concentrations of edelfosine (EDLF), and the ability to inhibit cell proliferation was measured after 72 h of treatment. The growth inhibition rate was calculated applying the GR metrics R package. The data represent the means ± SD of the GR metric scores. GR_50_ values for each pancreatic cancer primary culture are shown (inset). (**b**) Induction of apoptosis in human pancreatic cancer primary cultures by edelfosine treatment. PDAC087T, PDAC089T and PDAC082T were incubated with 10 and 20 μM edelfosine for the indicated incubation times, and the percentage of apoptosis was then determined by means of flow cytometry, as assessed by the increase of cells in the sub-G_0_/G_1_ region. Untreated control cells were run in parallel. The data are shown as the means ± SD of three independent experiments. (**c**) Effect of edelfosine on the proliferation capacity of CSC pancreatic cancer spheroids generated from PDAC082T, PDAC087T and PANC-1. The cells were incubated with different concentrations of edelfosine for 72 h. Growth inhibition was analyzed by using the GR metrics as above, and the GR_50_ values are shown. The results shown are the means ± SD of three independent experiments performed in triplicate.

A dose response curve was analyzed in the above PDAC-derived primary cell cultures, calculating the cell division rate by applying the growth rate inhibition (GR) metrics algorithm [53]. Interestingly, we observed a cell-dependent behavior, which was represented by the range of response to edelfosine (Figure 7a). PDAC082T was the most sensitive primary cell culture and displayed 50% growth inhibition (GR_50_) at ~0.1 μM, whereas PDAC076T showed the most resistant profile with a GR_50_ of 2.59 µM. PDAC087T and PDAC089T showed similar intermediate profiles with GR_50_ of 1.36 × 10^−6^ and 8.06 × 10^−7^ M, respectively (Figure 7a).

Edelfosine treatment also induced apoptosis in the patient-derived primary pancreatic cancer cell cultures, as assessed by an increase in the percentage of hypodiploid cells (sub-G_0_/G_1_ region), as a result of DNA breakage, measured by means of cell cycle analysis through flow cytometry (Figure 7b). The intensity of this proapoptotic activity of edelfosine on these primary cell cultures was cell-dependent, but, interestingly, it was not correlated with its capacity to affect cell proliferation (cf. Figure 7a,b). The data from Figure 7a indicated that PDAC082T cells were more sensitive than PDAC087T cells to inhibition of their cell proliferation and to undergoing cell death upon edelfosine treatment, whereas a higher apoptotic response was achieved in the latter cells (Figure 7b). This could be explained, at least in part, by the suggestion that edelfosine could promote an additional type of cell death different from apoptosis in PDAC082T cells. In this regard, we previously found that edelfosine induces necroptosis in apoptosis-reluctant glioblastoma cells [73]. Furthermore, the kind of cell death induced by edelfosine in different tumor cells highly depends on the cell type, the relative endogenous presence of different cell death-promoting molecules, as well as the activation of certain signal transduction pathways [73,74,75].

CSCs isolated from the above patient-derived primary pancreatic cancer cell cultures (~0.8 and ~0.3% CSCs in the PDAC082T and PDAC087T patient-derived primary pancreatic cancer cells), as mentioned above for PANC-1 cells, were sensitive to edelfosine, with GR_50_ values in the micromolar range (Figure 7c), being slightly more sensitive than the corresponding PANC-1 CSCs. Interestingly, CSCs generated from PDAC082T and PDAC087T rendered GR_50_ values that were rather similar to each other, although the GR_50_ value of the PDAC082T primary cell culture was 16 times lower than the corresponding value of the PDAC087T primary cell culture.

**Figure 8:**
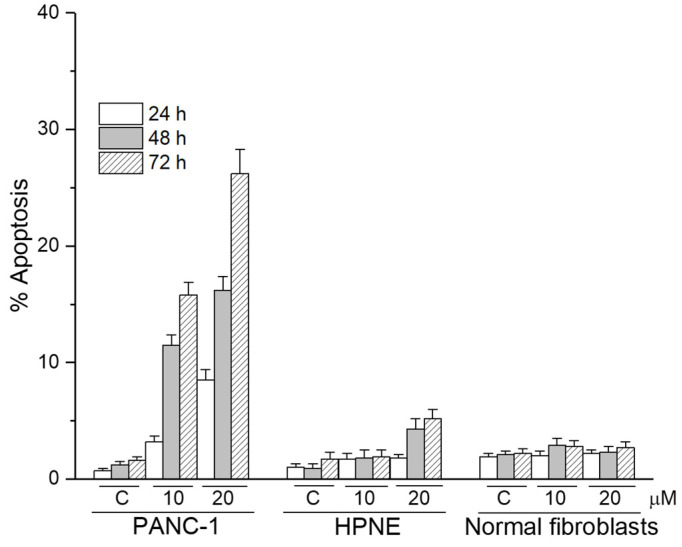
Differential capacity of edelfosine to induce apoptosis between pancreatic cancer cells and nontumorigenic cells. Human PANC-1 pancreatic cancer cells, nontumorigenic HPNE and normal fibroblasts show a different sensitivity to undergo apoptosis following treatment with edelfosine for the indicated incubation times and drug concentrations. The data are shown as the means ± SD of three independent experiments.

Unlike pancreatic cancer cells, nontumorigenic pancreatic HPNE cells as well as normal fibroblasts were rather resistant to the apoptotic action of edelfosine when used at 10 and 20 μM (Figure 8).

## 4. Discussion

We succeeded in isolating pancreatic CSCs from well-established human pancreatic cancer cell lines, such as the PANC-1 cell line, as well as from primary cultures of pancreatic cancer patients. These pancreatic CSCs show the following features: (a) cells are triple-positive for CD44, CD24 and EpCAM; (b) they have a high tumorigenic activity, developing tumors from implants of as few as 100 CSCs, displaying a 100-fold enhanced tumorigenic potential as compared to the parental pancreatic cancer population from which they derive; (c) they form CSC pancreatic cancer spheroids rapidly under a serum-free special cancer stem culture medium. Because the above pancreatic CSCs in culture keep approximately 3–7% triple-positive cells with time, it could be envisaged that these pancreatic CSCs are capable of undergoing both the processes of self-renewal and generation of a more differentiated progeny, thus having the ability to recapitulate the phenotype of the original tumor from which they are derived.

CSCs are rather refractory to most therapies and current drugs in clinical use [17,76,77], while most of the non-stem cell components of tumors could be eradicated. Thus, relapses and metastasis would be expected to occur if the drug-resistant CSC population remained intact, the latter being able to self-renew themselves and differentiate into heterogeneous lineages of cancer cells [17]. Here, we found for the first time that the antitumor ether lipid and alkylphosphocholine analog edelfosine, which shows a potent and selective antitumor activity in vitro and in vivo [26,27,31,34,35,37], including in pancreatic cancer [29], is effective in getting rid of pancreatic CSCs. PANC-1 CSCs, as well as the CSCs derived from pancreatic cancer patients, were sensitive to edelfosine. Furthermore, edelfosine dramatically reduces the number and size of CSC colonies and pancreatic cancer spheroids, making them smaller, inhibiting their formation and proliferation, and inducing CSC apoptosis.

Regarding patient-derived primary pancreatic cancer cell cultures, there was a high variability in their sensitivity to edelfosine, but this variability was highly reduced when CSCs from each primary cell culture were isolated. Thus, PDAC082T cells showed a 16-fold lower GR_50_ than PDAC087T cells, but the corresponding GR_50_ values in the isolated CSCs from both primary cultures became similar each other, and resulted higher than those values corresponding to the primary cultures. Apparently, one could envisage that cells become more resistant to the antitumor ether lipid when a higher percentage of CSCs is present.

Interestingly, our results clearly reveal that edelfosine treatment elicits autophagy in pancreatic CSCs, likely as a protective and survival signal, and pretreatment with autophagy inhibitors highly potentiates the proapoptotic activity of this ether lipid against pancreatic CSCs.

Pancreatic cancer remains an incurable cancer for which no treatment is yet effective, and the survival rate has not improved in the last fifty years. Different approaches have been undertaken, including FOLFIRINOX and different combinations of gemcitabine, Abraxane, cisplatin, temsirolimus and bevacizumab [1,78], as well as synthesis of novel antitumor drugs, such as amphiphilic pyrrolidine derivatives [79] and isoprenylcysteine carboxyl methyltransferase inhibitors [80], but none has rendered significant outcomes in a clinical context so far. Here, we found that the alkylphospholipid analog edelfosine accumulates in the ER of different human pancreatic cancer cell lines [29] (this work) and in human pancreatic CSCs (this work), mounting a persistent ER stress response, as assessed by a dramatic and sustained CHOP upregulation, that eventually leads to apoptosis. Our data showed remarkable colocalization between fluorescent edelfosine and an ER marker, indicating that edelfosine accumulated in the ER of PANC-1 cells and PANC-1 CSCs. However, not all the cells underwent apoptosis after 3 or 5 days of drug treatment. This could be due to the fact that the induction of apoptosis would require the involvement of additional processes that need time to be triggered and completed, probably to exceed a certain drug concentration threshold in the subcellular organelle to initiate the next proapoptotic signaling, and/or the onset of a series of events that, once initiated in the ER, should be transmitted to mitochondria and other subcellular organelles before the eventual manifestation of the apoptotic response. In this regard, we previously found that edelfosine-induced apoptosis involves ER, mitochondria and intracellular trafficking to promote cell death [1,29,31,63,81,82], and there is a kind of connection between these processes [47,63,83] for the onset of cell death.

Interestingly, edelfosine also inhibits pERK survival signaling, which seems to play a major role in the triggering of autophagy as a protective response. In this regard, inhibition of the ERK/MAPK (mitogen-activated protein kinase) signaling downstream of KRAS in pancreatic cancer cells elicits autophagy as a survival response, in order to protect pancreatic cancer cells from the cytotoxic effects of ERK signaling inhibition [84]. This identifies autophagy as a promising Achilles heel for pancreatic cancer, and therefore novel approaches for the treatment of pancreatic cancer could involve the combined targeting of ERK signaling and autophagy [84,85,86].

The induction of apoptosis in pancreatic cancer cells, including pancreatic CSCs, through the onset of a persistent ER response opens up a new avenue for the treatment of pancreatic cancer, and highlights the ER as a major target in pancreatic cancer and pancreatic CSCs. On the other hand, a novel framework in cancer implies the involvement of neutrophils in cancer development and therapy [87]. In this context, recent data by our group indicate that pancreatic cancer cells are particularly sensitive to human neutrophil arginase-mediated arginine deprivation, which leads to a strong ER stress response and potentiates the antitumor activity of the ER-targeting alkylphospholipid analog edelfosine [49].

On the other hand, a major problem in the treatment of pancreatic cancer is the overly high toxicity and adverse effects of the current therapeutic regimens [1]. However, edelfosine lacks significant toxicity, and histologic studies in rats showed no damage in the kidneys, liver, heart and stomach following edelfosine treatment [88].

It has been suggested that targeting CSCs can result in tumor elimination and prevention of tumor relapse [89]. Several recent reports have shown a number of drugs that affect the formation of pancreatic CSC spheres, such as doxycycline [90], bufalin [91], resveratrol [92] and the small anticancer molecule FL118 [93], but the data reported here provide novel aspects that could lead to the elimination of CSCs, thus favoring a new framework in the search of a cure for this dismal cancer. The ether lipid edelfosine accumulates in the ER of pancreatic CSCs, which constitutes a hallmark in pancreatic cells, and therefore could be a promising therapeutic target to be used to mount a persistent ER stress response, leading eventually to apoptosis [1]. Edelfosine can induce apoptosis in pancreatic CSCs, and this tumor-killing activity is potentiated by autophagy inhibitors, such as chloroquine. Its oral administration, lack of significant toxicity and ease of handling make this alkylphospholipid analog a promising drug candidate for further development and clinical studies.

## 5. Conclusions

The antitumor ether lipid edelfosine induces apoptosis in CD44^+^CD24^+^EpCAM^+^ pancreatic cancer stem cells (CSCs) and blocks the formation of pancreatic CSC spheroids, derived from the established human pancreatic cancer cell lines as well as from pancreatic cancer patient-derived primary cultures. Edelfosine accumulates in the endoplasmic reticulum (ER) of pancreatic CSCs, leading to persistent ER stress and eventually to cell death. Autophagy is triggered in CSCs as a protective signal to ER stress following edelfosine treatment, and thereby the combination of endoplasmic reticulum targeting with the action of autophagy inhibitors potentiates the proapoptotic activity of edelfosine against CSCs, and represents a promising approach to pancreatic cancer therapy. In addition, orally administered edelfosine lacks significant toxicity, and hence further studies are warranted to unveil its potential as a promising new approach in pancreatic cancer chemotherapy.

## Figures and Tables

**Table 1:** Tumor formation ability of sorted CD44^+^CD24^+^EpCAM^+^ pancreatic CSCs isolated from the human PANC-1 pancreatic cell line.

Marker	Number of Cells		
	10^2^	10^3^	10^4^
Unsorted PANC-1	0/6 (0%)	2/6 (33.3%)	4/6 (66.6%)
CD44^+^CD24^+^EpCAM^+^ PANC-1 CSCs	6/10 (60%)	10/10 (100%)	10/10 (100%)

The cells were isolated by means of flow cytometry and sorted for CD44^+^CD24^+^EpCAM^+^ cells. Sorted triple-positive cells were assayed for the ability to generate tumors after a subcutaneous injection into the flank of SCID mice at 10^2^, 10^3^ and 10^4^ cells per injection. The mice were examined for tumor formation by palpation and subsequent autopsy during the following 17 weeks after injection. The data are shown as the number of mice that developed tumors/number of animals undergoing cell injection. The percentage of the mice that developed tumors is indicated in parentheses.

**Table 2:** Effect of edelfosine on the formation of pancreatic CSC spheroid colonies.

Treatment	Perimeter (AU)	*p*	Spheroid Colony Numbers	*p*
Control	5.46 ± 2.58		145 ± 18	
1 μM EDLF	4.01 ± 2.20	0.25	127 ± 6	0.1013
5 μM EDLF	1.95 ± 0.73	0.0005	70 ± 12	0.0004
10 μM EDLF	1.46 ± 0.70	0.0001	46 ± 13	0.0001
20 μM EDLF	1.44 ± 0.60	0.0002	32 ± 7	<0.0001

Data are from the experiments similar to those depicted in Figure 3. Statistical significance is relative to untreated controls. Perimeter is measured in arbitrary units. The number of tumor spheres in each plate was counted under a stereo microscope. The results are shown as the means ± SD of at least three independent experiments. EDLF, edelfosine; AU, arbitrary units.

## Data Availability

Data are contained within the article or supplementary material. The data presented in this study are available on request from the corresponding author.

## Supplementary Materials

The following are available online at https://www.mdpi.com/article/10.3390/cancers13236124/s1. Figure S1: Original and uncropped images supporting the gel results corresponding to Figure 4b, Figure 5b and Figure 6a–c; Figure S2: Characterization of primary pancreatic cancer cell cultures from patient-derived xenograft (PDX) models; Figure S3: Phase contrast photomicrographs of primary cell cultures showing homogenous epithelial morphology.
