# Supplementary material for: Induction of Apoptosis in Human Pancreatic Cancer Stem Cells by the Endoplasmic Reticulum-Targeted Alkylphospholipid Analog Edelfosine and Potentiation by Autophagy Inhibition
Source: Cancers (Basel). 2021 Dec 5;13(23):6124. doi: 10.3390/cancers13236124 (PMC8656492; doi:10.3390/cancers13236124)
Supplement: Supplementary file 1 [file cancers-13-06124-s001.zip › cancers-1485504-supplementary.pdf]

cancers

MDPI

Induction of Apoptosis in Human Pancreatic Cancer Stem Cells by the Endoplasmic Reticulum-Targeted Alkylphospholipid Analog Edelfosine and Potentiation by Autophagy Inhibition

Consuelo Gajate, Odile Gayet, Nicolas A. Fraunhoffer, Juan Iovanna, Nelson Dusetti and Faustino Mollinedo

Gajate et al.
Figure 4b

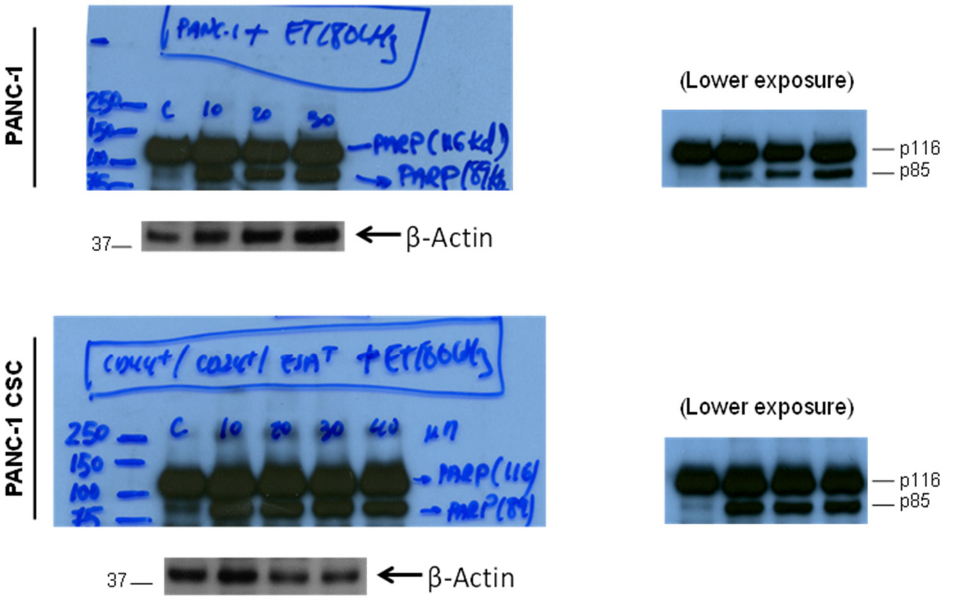

Gajate et al.
Figure 5b (PANC-1)

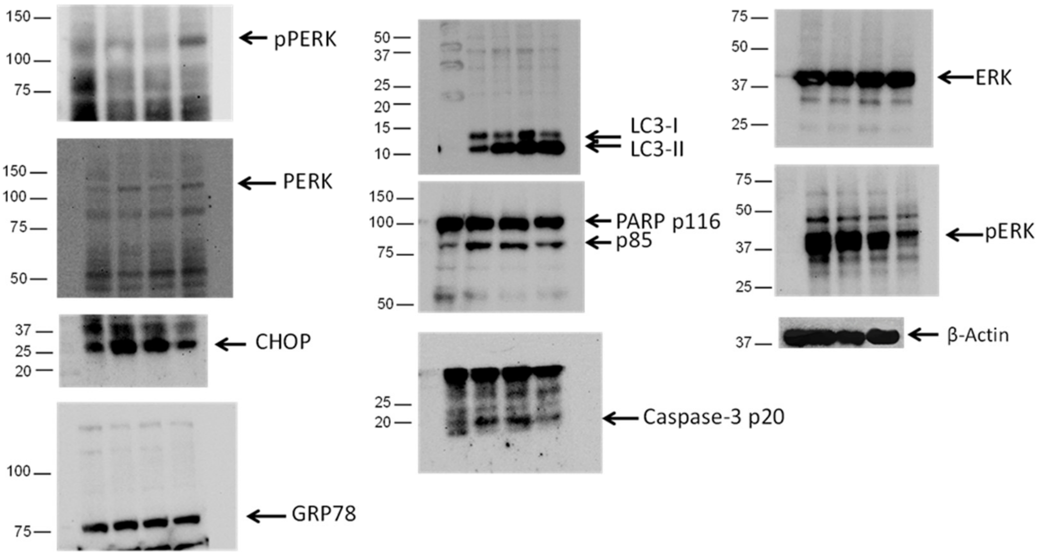

Gajate et al.
Figure 5b (PANC-1 CSC)

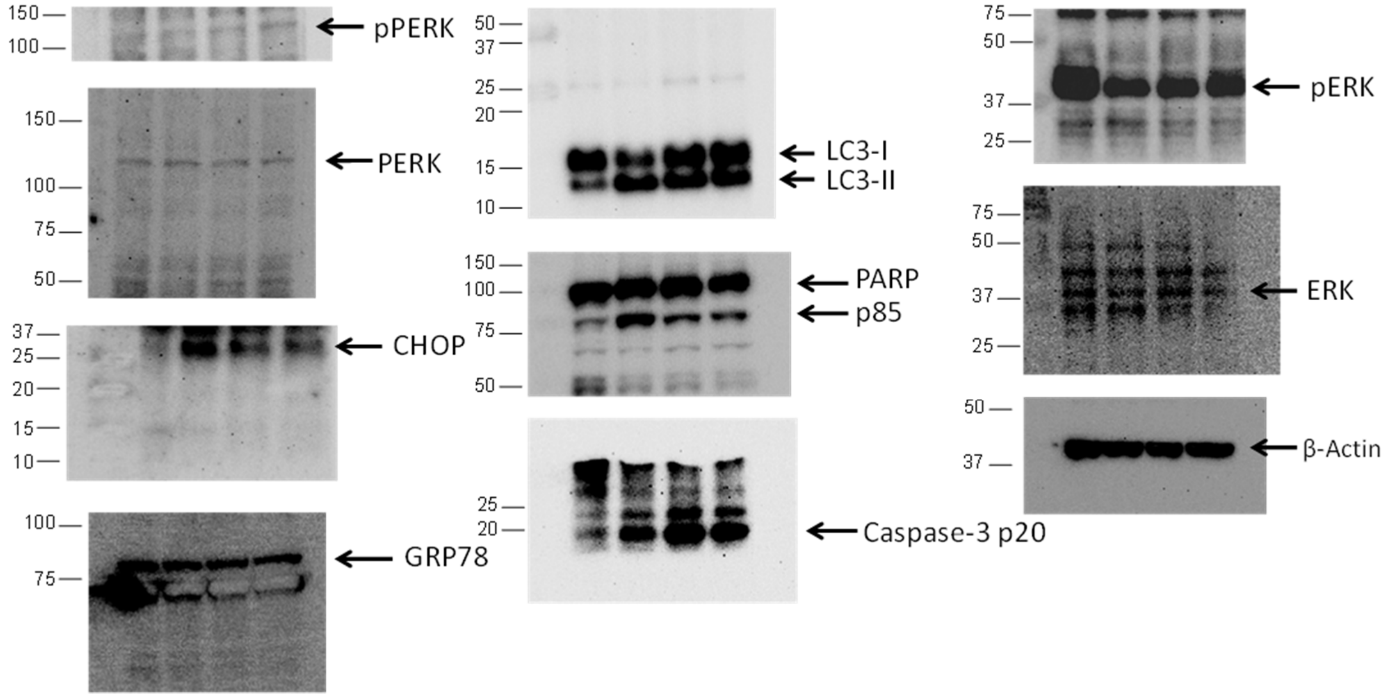

Gajate et al.
Figure 6a

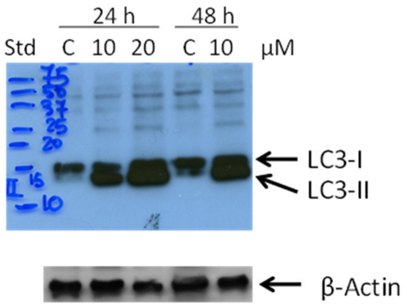

Std – Protein molecular weight standards
C – Control untreated cells

Gajate et al.
Figure 6b

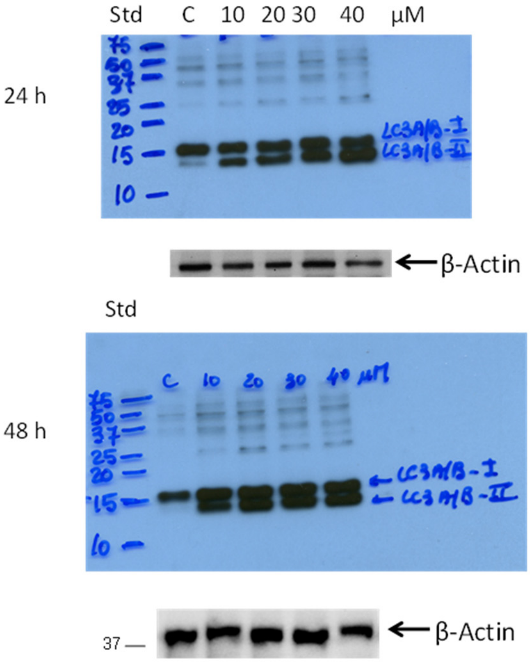

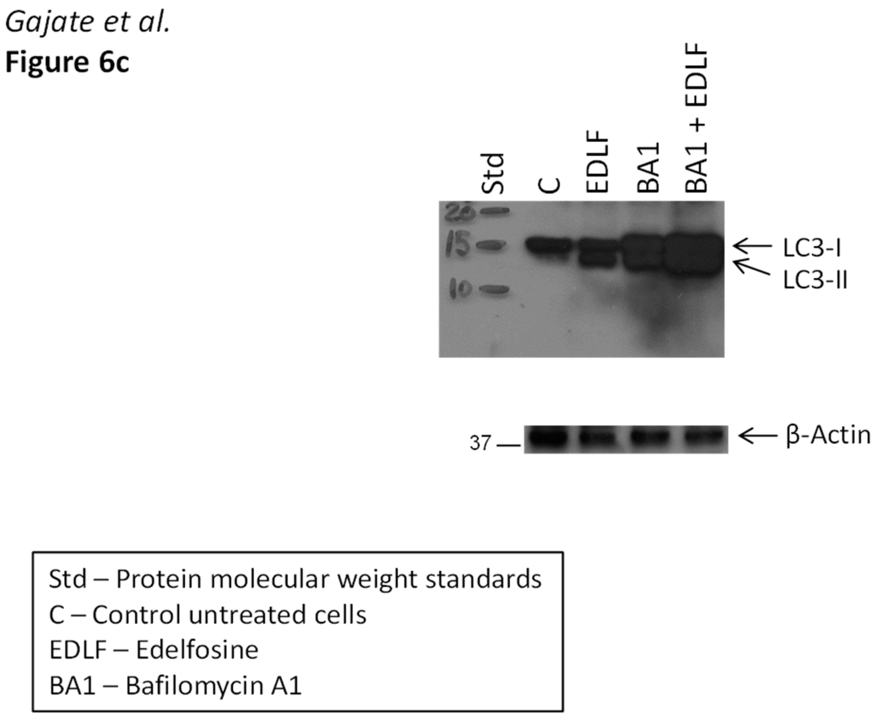

Figure S1. Original and uncropped images supporting the gel results corresponding to Figure 4b, Figure 5b and Figure 6a-c. Chemiluminescence from original blots corresponding to Figure 4b, Figure 5b and Figure 6a-c was detected by using a ChemiDoc Imaging System (Bio-Rad) (Fig. 5b) or X-ray autoradiography (Fig. 4b and Fig. 6a-c). Some blots were already cut in strips, corresponding to the approximate molecular weight of the protein of interest, before incubation with the primary antibody to identify different proteins. β-Actin was always identified in blots cut in strips corresponding to the approximate region of 42 kDa.

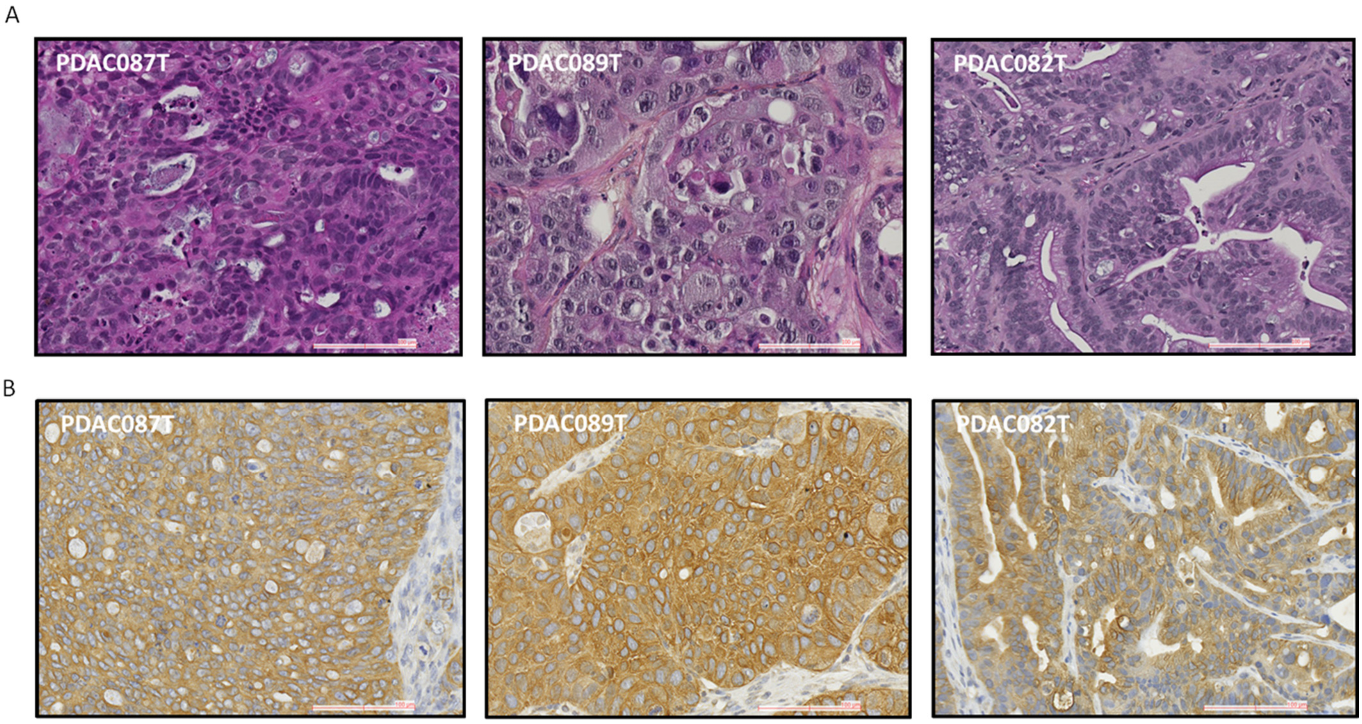

Figure S2. Characterization of primary pancreatic cancer cell cultures from patient-derived xenograft (PDX) models. (A) Hematoxylin-eosin staining of PDX derived from subcutaneously injected primary cell cultures. (B) Cytokeratin 19 immunostaining of the PDX models indicating human epithelial origin of stained cells. Human cells are stained in brown.

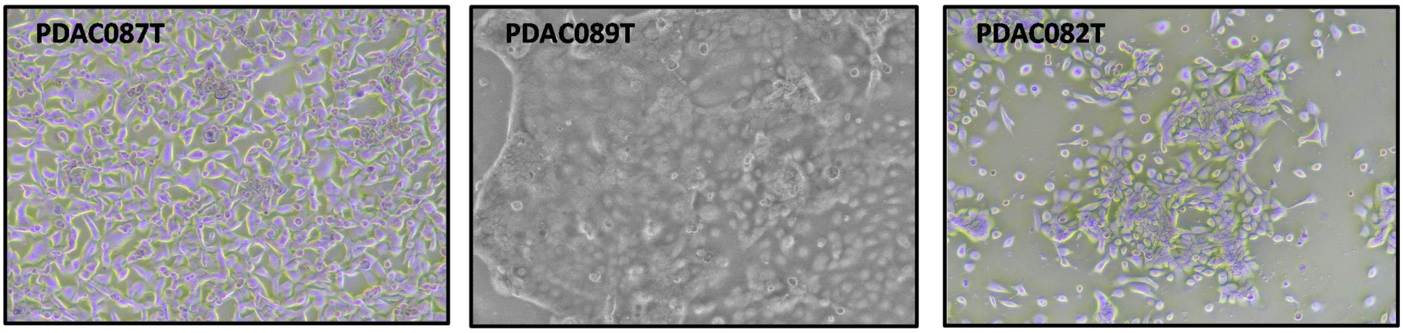

Figure S3. Phase contrast photomicrographs of primary cell cultures showing homogenous epithelial morphology.
